# Cell Adhesion Signaling and Its Impact on Tumorigenesis

**DOI:** 10.1155/2010/257809

**Published:** 2011-01-11

**Authors:** Claudia D. Andl, Ala-Eddin Al Moustafa, Therese B. Deramaudt, Geraldine M. O'Neill

**Affiliations:** ^1^Vanderbilt University, Nashville, TN, USA; ^2^Syrian Research Cancer Centre of the Syrian Society against Cancer, Aleppo, Syria; ^3^McGill & Concordia Universities, Montreal, Canada; ^4^Universite de Strasbourg, CNRS UMR 7213, Illkirch, France; ^5^Kids Research Institute, The Children's Hospital at Westmead, Sydney, NSW 2145, Australia; ^6^Discipline of Paediatrics and Child Health, The University of Sydney, Sydney, NSW 2006, Australia

Cell adhesion can be divided into cell-cell adhesion mediated by three major structures, tight junctions, adherens junctions, desmosomes, and cell-matrix adhesion, which is for the most part integrin-based. Cell-cell interactions play an important role in the maintenance and integrity of tissues and organs but similar to cell-matrix complexes may also transduce cell signaling. While this is the basics for understanding cell adhesion and its function, the role of cell adhesion proteins in disease, especially tumorigenesis, is far more diverse. A summary is given in the paper presented by Dr. Andl in this issue where she also focuses on recently discovered cell adhesion components and the regulation of cell adhesion molecules by miRNAs. In cancer, cell adhesion molecules are generally thought to be deactivated in order to aid in cancer cell dissemination and dispersal. However, aside from the loss of function, various gain-of-function events occur during induction and progression of cancer that can be mediated by cell adhesion molecules. Many of the molecules involved in these processes, such as the components of desmosomes, were initially identified as targets of autoimmune diseases. This special issue outlines the latest findings in research and review articles.

Tight junctions mediate apical cell-cell adhesion and regulate epithelial cell polarity. Members of the claudin family are involved in a number of diseases including cancer; however, little is known regarding the mechanisms behind their pathophysiological function. Drs. Singh, Sharma, and Dhawan give an overview of the current state of knowledge in this issue. To identify cell transformation mechanisms other than previously described, Lynch et al. investigated the effect of MMP-7 mediated cleavage of E-cadherin. They demonstrate that loss of cell-cell adhesion is induced following E-cadherin processing, which results in increased cell migration, loss of polarization, and activation of RhoA.

Interactions of cell adhesion molecules with their environment are central to their regulation and to cell migration and invasion. In the tumor context, the array of integrin receptors expressed causes the cells to differentially respond to external signals, resist the effects of cytotoxic drugs, and facilitate growth and invasion through a range of different tissue environments encountered by invasive cancer cells. In particular, the regulation of integrin receptor interaction with the extracellular matrix is thought to be critical for those cancers that invade and metastasize using a mesenchymal mode of migration. Jossen et al. show a novel prostate cancer bone metastatic model to assay drug sensitivity in three-dimensional cultures that mimic the preclinical or clinical setting more accurately. In here, they show that prostate cells revert to more epithelial cells, mesenchymal-epithelial transition (MET) in the presence of bone stroma modeling later stages of metastatic colonization, and are sensitized to radiation treatment when blocking E-cadherin or *α*-integrin. Mesenchymal migration as a therapeutic target for glioblastoma is discussed by Zhong et al. Another potential therapeutic agent, a Src inhibitor, is described by Yaseem et al. Comparison of SKI-606 and Iressa effects, Src/Abl and EGFR inhibitors, respectively, on cervical cancer cell lines shows inhibition of cell proliferation. Additionally, SKI-606 decreases cell migration and invasion through MET accompanied by the re-expression of E-cadherin and the recruitment of *β*-catenin to the cell membrane. Hironobu Yamashita et al. demonstrate that lysophosphatidic acid, a phospholipid growth factor, induces the stimulation of lamellipodia formation and enhanced cell migration through the upregulation of the TGF*β*1 target gene Laminin-322. The role of focal adhesion kinase (FAK) phosphorylation in the dynamic regulation of integrin-based adhesions and cellular migration is shown in a paper by Hamadi et al. Upon pervanadate-induced phosphorylation, FAK delocalizes from focal adhesions to newly formed membrane ruffles in a src-dependent event. Finally, advances in nanotechnology that allow the study of cellular transformation in engineered cellular environments are introduced by Siti Hawa Ngalmi et al. Such cross-disciplinary approaches have begun to advance our knowledge of how adhesion signaling is organized and how cells relay information regarding the composition and structure of the external environment into biological activity. In particular, these advances are being driven by novel microscopy technologies and the continuing development of new techniques that allow us to visualize the intricate workings of adhesion-dependent signaling processes.

This synopsis gives a glimpse at the current leading edge science published in this special issue of the Journal of Oncology. We hope that these papers inspire interest in multidisciplinary approaches as well as in the identification of novel therapeutics.

*Claudia D. Andl* *Claudia D. Andl* *Ala-Eddin Al Moustafa* *Ala-Eddin Al Moustafa* *Therese B. Deramaudt* *Therese B. Deramaudt* *Geraldine M. O'Neill* *Geraldine M. O'Neill*

